# The Risk of Type 2 Diabetes and Coronary Artery Disease in Non-obese Patients With Non-alcoholic Fatty Liver Disease: A Cohort Study

**DOI:** 10.3389/fcvm.2021.680664

**Published:** 2021-08-20

**Authors:** Wen Dai, Ziyu Zhang, Shuiping Zhao

**Affiliations:** Department of Cardiology, The Second Xiangya Hospital, Central South University, Changsha, China

**Keywords:** non-alcoholic fatty liver disease, coronary artery disease, non-obesity, risk factor 2, diabetes

## Abstract

**Background:** Non-alcoholic fatty liver disease (NAFLD) is not uncommon in non-obese subjects, referred to as non-obese NAFLD. It is not fully determined whether non-obese NAFLD is associated with increased risks of type 2 diabetes (T2D) and coronary artery disease (CAD) in Chinese. This study aimed to examine the association between NAFLD and risks of T2D and CAD in a non-obese Chinese population.

**Methods:** The present cohort study included two stages. In the first cross-sectional study, 16,093 non-obese subjects with a body max index (BMI) < 25.0 kg/m^2^ were enrolled from The Second Xiangya Hospital, China, from 2011 to 2014. Hepatic steatosis was evaluated by ultrasonography examination. Logistic regression analyses were used to examine the association of non-obese NAFLD with T2D and CAD at baseline. In the subsequent 5-year follow-up study, 12,649 subjects free of T2D and CAD at baseline were included, and the incidence of T2D and CAD were observed. Cox proportional hazard regression analyses were performed to determine the risk of incident T2D and CAD with NAFLD.

**Results:** At baseline, the prevalence of NAFLD, T2D and CAD were 10.7% (1,717/16,093), 3.3% (529/16,093) and 0.7% (113/16,093), respectively. The univariate logistic regression analyses showed NAFLD associated with both T2D and CAD. Moreover, in a multivariate logistic regression model, NAFLD remained independently associated with T2D (OR: 2.7, 95% CI: 2.2–3.3, *p* < 0.001). However, no significant association was found between NAFLD and CAD by the multivariate logistic regression analyses (OR: 1.1, 95% CI: 0.6–1.8, *p* = 0.854). During a 5-year follow-up period, 177 (1.4%) patients developed T2D, and 134 (1.1%) developed CAD, respectively. In univariate Cox regression models, NAFLD associated with both T2D and CAD. Moreover, the multivariate Cox regression analysis revealed that NAFLD independently associated with an increased risk of T2D (HR: 2.3, 95% CI: 1.7–3.2, *p* < 0.001). However, the association between NAFLD and incident CAD was lost in the multivariate Cox regression analysis (HR = 1.5, 95% CI: 1.0–2.4, *p* = 0.059).

**Conclusions:** NAFLD was an independent risk factor for T2D in non-obese subjects. However, no significant association was observed between non-obese NAFLD and incident CAD after adjusting other traditional cardiovascular risk factors, suggesting these factors might mediate the increased incidence of CAD in non-obese NAFLD patients.

## Introduction

Non-alcoholic fatty liver disease (NAFLD) is the hepatic manifestation of metabolic syndrome. It refers to a group of conditions with the accumulation of excess fat in the liver while excluding other known etiologies, such as significant alcohol consumption and viral hepatitis (1, 2). Histologically, NAFLD encompasses a continuum of severity ranging from isolated steatosis (more than 5% of fatty infiltration of hepatocytes) to significant non-alcoholic steatohepatitis (NASH), characterized by fatty infiltration plus varying degrees of necroinflammation and/or fibrosis, and eventually to cirrhosis and hepatocellular carcinoma (1, 3). The prevalence of NAFLD has dramatically increased over past decades mostly due to the obesity pandemic. It is estimated that ~25% of the population worldwide is affected by NAFLD (4). NALFD increases the risk of hepatocyte injury and even liver failure. NAFLD is the most common cause of elevated liver chemistry test results (2, 5). NASH has become the leading cause of liver failure and a major indication for liver transplantation (6–9).

Moreover, NAFLD is also closely associated with some extrahepatic implications, such as type 2 diabetes (T2D) and cardiovascular disease (CVD) (10). A strong association between NAFLD and T2D has been shown, as more than 70% of patients with T2D have NAFLD (11–13). Patients with NAFLD had an increased risk of CVD (14). Compared with the general population, patients with NAFLD, particularly NASH, have a reduced survival rate mainly attributed to CVD (2, 15, 16). Obesity is the major risk factor for NAFLD and could also contribute to the increased risk of T2D and CVD in NAFLD (17).

However, NAFLD is also found in non-obese individuals, often referred to as non-obese NAFLD (18, 19). The pathogenesis of non-obese NAFLD is not well-known. The prevalence of non-obese NAFLD ranges widely from 3 to 30% (19), and it has been reported that the prevalence in Asia is higher than in Western countries (20). The variation suggests that genetic factors may be critical in the pathogenesis of non-obese NAFLD. Increasing interests are gained on the metabolic consequences of non-obese NAFLD. However, very little data from prospective studies are available about the risk of incident T2D and coronary artery disease (CAD) in the Chinese population, and ethnic variations might exist in the association of non-obese NALFD with T2D and CAD. Therefore, in this study, we aimed to investigate the association of NAFLD with the risk of T2D and CAD in a large size non-obese Chinese population.

## Methods

### Study Design and Population

This study was designed as an observational cohort study, which included two stages. The first was a cross-sectional study to examine the association of non-obese NAFLD with T2D and CAD at baseline. When data of this stage were obtained, we then conducted a 5-year follow-up study of baseline T2D and CAD-free subjects to determine the association of non-obese NAFLD with the risk of incident T2D and CAD.

**Figure 1:**
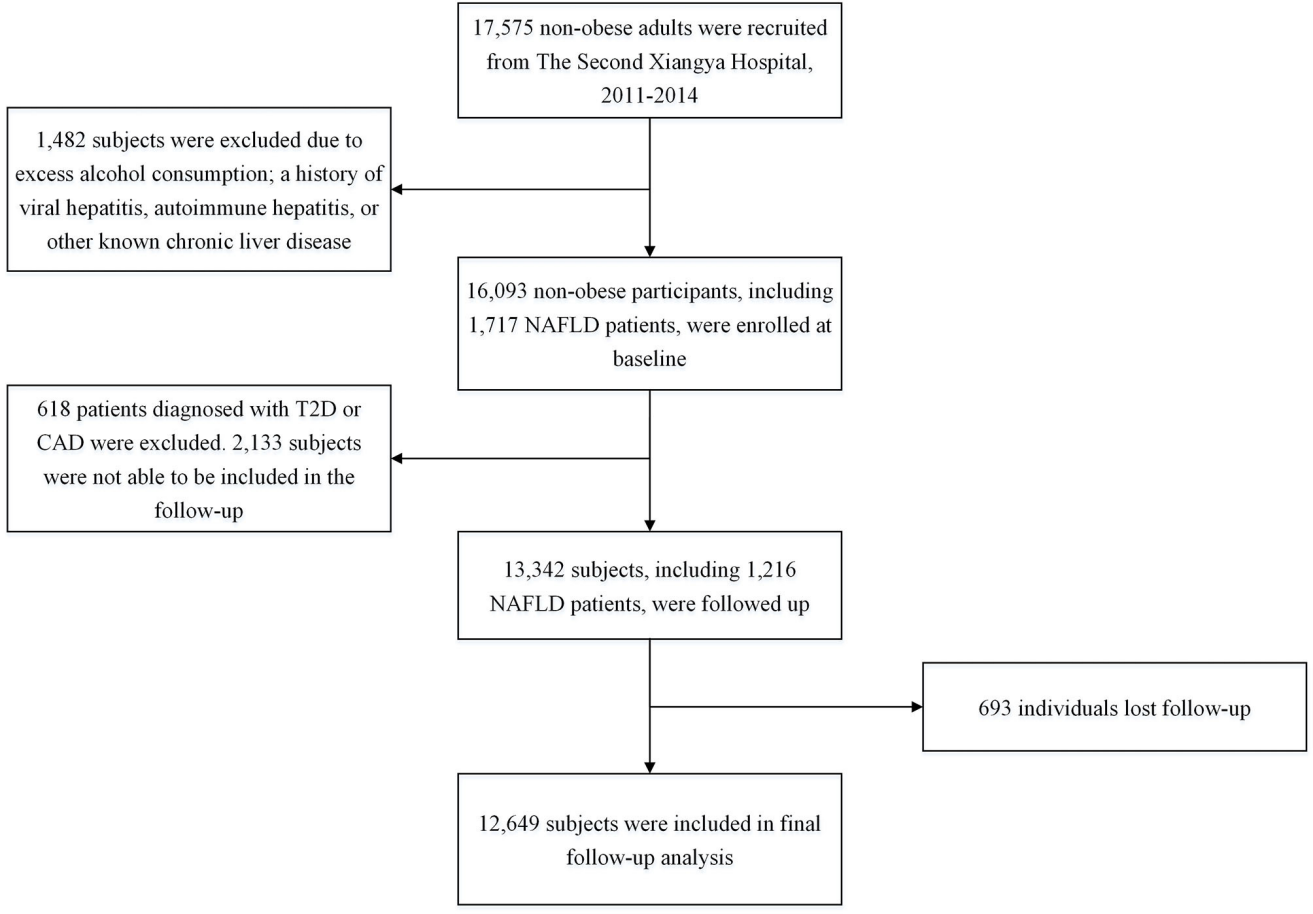
Flowchart of the study population selection. NAFLD, Non-alcoholic fatty liver disease; T2D, type 2 diabetes; CAD, coronary artery disease.

The process of study population sample selection was described in Figure 1. Initially, 17,575 adult non-obese subjects were consecutively recruited from The Second Xiangya Hospital, Central South University, Changsha, China, from 2011 to 2014. Body mass index (BMI) < 25.0 kg/m^2^ signifies non-obesity in Chinese population (17). Subsequently, 1,482 subjects were excluded due to excess alcohol consumption, a history of viral hepatitis, autoimmune hepatitis, or other known chronic liver disease. Therefore, 16,093 non-obese participants were enrolled in the first cross-sectional study. Of which, 1,717 patients were diagnosed with NAFLD. Besides, 539 subjects were identified to have T2D and 113 patients were found to have CAD.

In the subsequent follow-up study, of 16,093 participants at baseline, 618 patients diagnosed with T2D or CAD were excluded. Besides, 2,133 subjects were not able to be included in the follow-up, and 693 individuals lost follow-up. Eventually, 12,649 subjects were included in the final analysis. The study was carried out following the 1975 Declaration of Helsinki and pertinent regulations. This study was approved by the ethics committee institutional review board of The Second Xiangya Hospital of Central South University. Informed written consent was obtained from all participants.

### Baseline Examinations

Clinical examinations were performed at baseline. Data of demographic characteristics, medical histories, anthropometric measurements, blood sample measurements, and hepatic ultrasonic examination were collected. NAFLD was diagnosed based on the evidence of fatty liver upon hepatic ultrasonography while excluding other known causes (4). Moreover, the severity of fatty liver on ultrasonography was graded as mild, moderate, and severe according to criteria as previously (21, 22). In brief, mild fatty liver is defined as slight diffuse increase in fine echoes in the liver parenchyma with normal visualization of the diaphragm and intrahepatic vessel borders. Moderate fatty liver is defined as moderate diffuse increase in fine echoes with slightly impaired visualization of the intrahepatic vessels and diaphragm. Severe fatty liver is defined as marked increase in fine echoes with poor or no visualization of the intrahepatic vessel borders, diaphragm and posterior portion of the right lobe of the liver. Hypertension was considered blood pressure ≥ 140/90 mmHg in more than two measurements and/or the requirement of or treatment with anti-hypertension drugs (23). At least 12-h overnight fasting blood samples were taken from participants. All laboratory parameters were measured at the Central Laboratory in The Second Xiangya hospital, which is standardized and certified. Platelets were counted by an automated blood cell analyzer. Hemoglobin A1c (HbA1c) was evaluated by the Tosoh Automated Glycohemoglobin Analyser (HLC-723G8, Tokyo, Japan). Other blood biochemistry profiles, including glucose, lipids, and liver and renal function tests, were analyzed by an automatic biochemistry analyzer (Hitachi 7360; Hitachi Ltd., Tokyo, Japan) with standard methods. The hepatic ultrasonic examination was carried out using a Toshiba Nemio 20 sonography machine (Toshiba, Tokyo, Japan).

### Follow-Up and Outcome Evaluations

Participants were followed up annually by clinic visits during the observation period. T2D and CAD status were evaluated by physicians who were blinded to study design and previous data. T2D was defined as the existence of fasting blood glucose levels ≥ 7.0 mmol/L, or 2-h postprandial blood glucose ≥ 11.1 mmol/L on the oral glucose tolerance test, or HbA1c level ≥ 6.5%, or the requirement of hypoglycemic medications (24). Patients were diagnosed with CAD by at least two cardiologists via clinical evaluation according to recommendations (25–27). In brief, all participants in this study received the electrocardiogram (EKG) test. When a patient was suspected of CAD due to the clinical manifestation of chest pain and/or the potential myocardial ischemia suggested by EKG, advanced tests would be performed, such as exercise stress test and/or cardiac catheterization and angiogram. Based on the comprehensive analysis of those test results, the diagnosis of CAD would be concluded or excluded.

### Statistical Analysis

All statistical analyses were performed using SPSS software (version 22.0; SPSS Inc., Cary, Chicago, USA) and R language version 3.5.2 (Eggshell Igloo). Two-tailed *p*-values < 0.05 were considered statistically significant. Numerical variables were expressed as mean ± standard deviation (SD) or median (interquartile range) where applicable. Categorical variables were expressed as a percentage (number). Differences in numerical variables between groups were analyzed by the independent *t*-test or Mann-Whitney U test, as appropriate, and categorical variables were analyzed by the chi-square test or Fisher exact test. Logistic regression models were applied to perform association analyses of NAFLD with T2D and CAD at baseline. Cox proportional hazard regression analyses were used to evaluate the risk of incident T2D and CAD during the follow-up period with the presence of NAFLD. C-statistic and ΔC-statistic were calculated to evaluate the efficiency of models and the incremental predictive value of adding NAFLD status into the original model.

## Results

### Baseline Characteristics of the Study Population

A total of 16,093 non-obese subjects were enrolled at baseline, of which 45.8% (7,364/16,093) were males. Baseline characteristics of all participants were shown in Table 1. Their mean age was 43 (SD, 15) years, and their mean BMI was 21.7 (SD, 1.1) kg/m^2^. The prevalence of NAFLD in those non-obese subjects was 10.7% (1,717/16,093). Patients with NAFLD appeared to be older, male predominant, and had higher BMI, systolic and diastolic pressure, and levels of blood glucose, HbA1c, alanine aminotransferase (ALT), aspartate aminotransferase (AST), creatinine, uric acid, triglyceride (TG), total cholesterol (TC), low-density lipoprotein cholesterol (LDL-C) and non-high-density lipoprotein cholesterol (non-HDL-C), and lower high-density lipoprotein cholesterol (HDL-C) levels and AST/ALT ratios than those subjects without NAFLD (all *p* < 0.001) (Table 1). Besides, there was a higher prevalence of T2D, CAD, hypertension, and current smoking in the NAFLD compared to the non-NAFLD group (all *p* < 0.001) (Table 1). The prevalence of T2D and CAD were 11.9% (204/1,717) and 1.5% (25/1,717) in NAFLD group and were 2.3% (325/14,376) and 0.6% (88/14,376) in non-NAFLD group, respectively (Table 1). Moreover, the NAFLD group had higher medication percentages of anti-hypertension, anti-diabetes and lipid-lowering agents (all *p* < 0.001) (Table 1).

**Table 1:** Baseline characteristics of study population.

	**Total** **(** ***n*** **= 16,093)**	**NAFLD** **(** ***n*** **= 1,717)**	**Non-NAFLD** **(** ***n*** **= 14,376)**	***p***
**Clinical characteristics**				
Age, years	43 ± 15	51 ± 13	42 ± 15	<0.001
Male percentage, % (*n*)	45.8 (7,364)	78.8 (1,353)	41.8 (6,011)	<0.001
BMI, kg/m^2^	21.7 ± 2.0	23.6 ± 1.1	21.5 ± 2.0	<0.001
Systolic pressure, mm Hg	118 ± 16	128 ± 17	117 ± 16	<0.001
Diastolic pressure, mm Hg	73 ± 10	79 ± 10	72 ± 9	<0.001
Hypertension, % (*n*)	12.9 (2,083)	29.3 (503)	11.0 (1,580)	<0.001
T2D, % (*n*)	3.3 (529)	11.9 (204)	2.3 (325)	<0.001
CAD, % (*n*)	0.7 (113)	1.5 (25)	0.6 (88)	<0.001
Current smoking, % (*n*)	24.5 (3,945)	40.8 (700)	22.6 (3,245)	<0.001
**Biochemical parameters**				
Platelet, counts	210 ± 53	206 ± 54	211 ± 53	0.001
Blood glucose, mmol/L	5.2 ± 1.0	5.7 ± 1.6	5.1 ± 0.8	<0.001
HbA1c, %	5.5 ± 0.7	5.9 ± 1.1	5.4 ± 0.6	<0.001
ALT, U/L	18 (13–25)	26 (19–36)	17 (13–24)	<0.001
AST, U/L	20 (18–24)	22 (20–27)	20 (17–24)	<0.001
AST/ALT	1.1 (0.9–1.4)	0.9 (0.7–1.1)	1.2 (0.9–1.4)	<0.001
TP, g/L	73 ± 4	73 ± 4	73 ± 4	0.08
ALB, g/L	45 ± 3	45 ± 3	45 ± 3	0.197
GLB, g/L	28 ± 4	28 ± 4	28 ± 4	0.240
Total bilirubin, μmol/L	13.4 ± 5.4	13.7 ± 5.6	13.3 ± 5.4	0.018
Direct bilirubin, μmol/L	4.0 ± 1.7	3.9 ± 1.6	4.0 ± 1.8	0.008
BUN, mmol/L	4.9 ± 1.4	5.3 ± 1.4	4.8 ± 1.4	<0.001
Cr, μmol/L	72 ± 21	80 ± 18	71 ± 21	<0.001
UA, μmol/L	302 ± 82	360 ± 81	295 ± 80	<0.001
TC, mmol/L	4.82 ± 0.93	5.23 ± 1.06	4.77 ± 0.90	<0.001
LDL-C, mmol/L	2.76 ± 0.77	3.02 ± 0.87	2.72 ± 0.75	<0.001
Non-HDL-C, mmol/L	3.39 ± 0.93	4.02 ± 1.06	3.32 ± 0.88	<0.001
HDL-C, mmol/L	1.42 ± 0.32	1.21 ± 0.26	1.45 ± 0.32	<0.001
TG, mmol/L	1.04 (0.76–1.49)	1.77 (1.25–2.58)	0.98 (0.74–1.37)	<0.001
**Medication**				
Anti-hypertension agents, % (*n*)	11.9 (1,919)	27.2 (467)	10.1 (1,452)	<0.001
Lipid-lowering agents, % (*n*)	19.4 (3,122)	32.0 (549)	17.9 (2,573)	<0.001
Anti-diabetes agents, % (*n*)	2.7 (438)	10.4 (179)	1.8 (259)	<0.001

*Data were shown as mean (standard deviation), median (Q1–Q3 quartiles), or number (percentage) as appropriate. P-values from analyses of the independent t-test, Mann-Whitney U test, or chi-square test for comparisons between NAFLD and non-NAFLD group. Two-tailed p < 0.05 was considered statistically significant. NAFLD, Non-alcoholic fatty liver disease; BMI, body mass index; AST, aspartate aminotransferase; ALT, alanine aminotransferase; TP, total protein; ALB, albumin; GLB, globulin, BUN, blood urea nitrogen; Cr, creatinine; UA, uric acid; TC, total cholesterol; LDL-C, low density lipoprotein cholesterol; HDL-C, high density lipoprotein cholesterol; non-HDL-C, non-high density lipoprotein cholesterol; TG, triglyceride*.

### Association of Non-obese NAFLD With T2D and CAD at Baseline

Logistic regression models were used to examine the association at baseline of NAFLD with T2D and CAD, respectively. Initially, univariate analyses showed NAFLD associated with both T2D and CAD [odds ratio (OR): 5.8, 95% confidence interval (CI): 4.9–7.0, *p* < 0.001 for T2D; OR: 2.4, 95% CI: 1.5–3.8, *p* < 0.001 for CAD] (Table 2). Furthermore, in a multivariate model adjusting factors of age, gender, BMI, and history of current smoking, NAFLD remained independently associated with T2D (OR: 2.7, 95% CI: 2.2–3.3, *p* < 0.001). However, no significant association was found between NAFLD and CAD in the multivariate model controlling variables of age, gender, BMI, LDL-C, HDL-C, TG, creatinine, and the history of T2D, hypertension, smoking, and medication (OR: 1.1, 95% CI: 0.6–1.8, *p* = 0.854) (Table 2).

**Table 2:** Logistic regression analyses of the association of NAFLD with T2D and CAD disease at baseline.

	**Univariate analysis**			Multivariate analysis^*^		
**Variables**	**OR**	**95% CI**	***p***	**OR**	**95% CI**	***p***
T2D	5.8	4.9-7.0	<0.001	2.7	2.2–3.3	<0.001
CAD	2.4	1.5-3.8	<0.001	1.1	0.6–1.8	0.854

* *Multivariate model adjusting for age, gender, body mass index and smoking for the OR of T2D; multivariate model adjusting for age, gender, body mass index, smoking, hypertension, T2D, low density lipoprotein cholesterol, high lipoprotein cholesterol, triglyceride, creatinine and medication history for the OR of CAD. P-values from logistic regression analyses. Two-tailed p < 0.05 was considered statistically significant. NAFLD, Non-alcoholic fatty liver disease; T2D, type 2 diabetes; CAD, coronary artery disease; OR, odds ratio; CI, confidence interval*.

### Incidence of T2D and CAD During Follow-Up

A total of 12,649 subjects, consisting of 5,706 males and 6,943 females free of T2D and CAD at baseline, were included for the final follow-up analysis. Of which, 1,216 patients had NAFLD. During the 5-year follow-up, 177 (1.4%) patients developed T2D, and 134 (1.1%) developed CAD, respectively. Furthermore, the incidence of both T2D and CAD was significantly higher in NAFLD relative to the non-NAFLD group (NAFLD vs. non-NAFLD: 4.8 vs. 1.0% for T2D, 2.3 vs. 0.9% for CAD, both *p* < 0.001) (Supplementary Table 1).

### Non-obese NAFLD and Risk of Incident T2D and CAD

**Figure 2:**
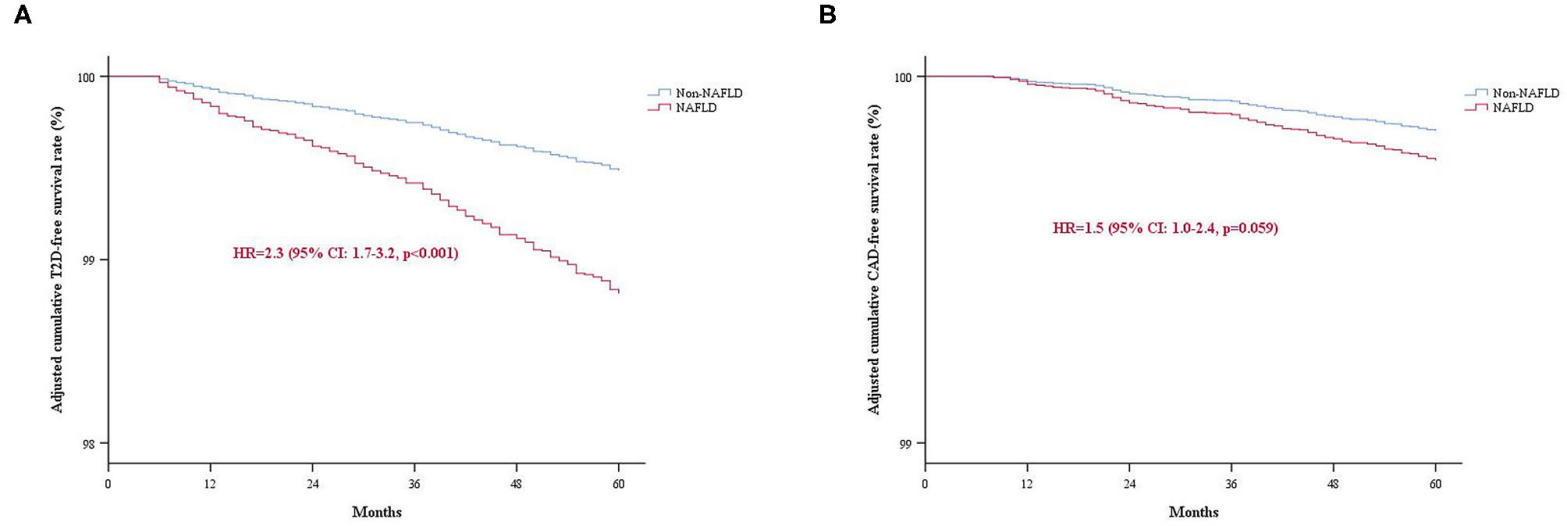
The adjusted cumulative events-free survival rate of the study population categorized by NAFLD. **(A)** T2D-free survival rate; **(B)** CAD-free survival rate. NAFLD, Non-alcoholic fatty liver disease; T2D, type 2 diabetes; CAD, coronary artery disease.

Cox proportional hazard regression analyses were performed to determine the risk of incident T2D and CAD during follow-up with NAFLD. In univariate models, NAFLD associated with both T2D [hazard ratio (HR): 4.7, 95% CI: 3.4–6.4, *p* < 0.001] and CAD (HR: 2.5, 95% CI: 1.6–3.8, *p* < 0.001) (Table 3). After adjusting for potential confounders including age, gender, BMI, and current smoking, the multivariate analysis revealed that NAFLD independently associated with an increased risk of T2D (HR: 2.3, 95% CI: 1.7–3.2, *p* < 0.001). However, the association between NAFLD and CAD risk was lost in a multivariate model with adjustment for age, gender, BMI, LDL-C, HDL-C, TG, creatinine, and status of hypertension and smoking (HR: 1.5, 95% CI: 1.0–2.4, *p* = 0.059). The adjusted cumulative T2D or CAD-free survival curve were shown in Figure 2. Moreover, we evaluated the value of NAFLD in predicting the risk of incident T2D by calculating C-statistic and ΔC-statistic. The C-statistic value was 0.850 (95% CI: 0.830–0.871) for an original prediction model, including factors of age, gender, BMI, and smoking. Moreover, the value increased to 0.860 (95% CI: 0.841–0.880) for the model further added with the variable of NAFLD status (ΔC-statistic: 0.010, 95% CI: 0.002–0.020, *P* = 0.015) (Supplementary Table 2).

**Table 3:** Cox proportional hazard regression analyses of the association of NAFLD with risks of T2D and CAD during follow-up.

		**Univariate analysis**			Multivariate analysis^*^	
	**HR**	**95% CI**	***p***	**HR**	**95% CI**	***p***
T2D	4.7	3.4-6.4	<0.001	2.3	1.7–3.2	<0.001
CAD	2.5	1.6-3.8	<0.001	1.5	1.0–2.4	0.059

* *Multivariate model adjusting for age, gender, body mass index and smoking for the HR of T2D; multivariate model adjusting for age, gender, body mass index, smoking, hypertension, low density lipoprotein cholesterol, high lipoprotein cholesterol, triglyceride and creatinine for the HR of CAD. P-values from Cox proportional hazard regression analyses. Two-tailed p < 0.05 was considered statistically significant. NAFLD, Non-alcoholic fatty liver disease; T2D, type 2 diabetes; CAD, coronary artery disease; HR, hazard ratio; CI, confidence interval*.

Moreover, we examined the association of NAFLD severity with T2D and CAD risk. We found that NAFLD independently associated with incident T2D while did not associate with CAD risk in the non-obese participants during the 5-year follow-up, regardless of NAFLD severity (Supplementary Table 3). The severity of fatty liver was graded as mild (*n* = 1,064, 87.5%), moderate (*n* = 147, 12.1%), and severe (*n* = 5, 0.4%) on ultrasonography. Moderate and severe NAFLD were combined into a moderate to severe NAFLD category for analyses, owing to the small number of severe NAFLD. The HR for T2D was 2.4 (95% CI: 1.7–3.3, *p* < 0.001) with mild NAFLD and was 2.1 (95% CI: 1.0–4.4, *p* = 0.049) with moderate to severe NAFLD. Moreover, the HR for CAD was 1.5 (95% CI: 1.0–2.5, *p* = 0.071) with mild NAFLD and was 1.5 (95% CI: 0.5–4.2, *p* = 0.429) with moderate to severe NAFLD.

## Discussion

In the current study, initially, we found that NAFLD independently associated with T2D at baseline in a large size non-obese population. Furthermore, during a 5-year follow-up period, we demonstrated that NAFLD was independently associated with an increased risk of incident T2D. Moreover, the addition of NAFLD status into the model improved the risk prediction for T2D in the non-obese population. However, no significant associations were observed between NAFLD and CAD both at baseline and during the follow-up period after adjusting for other potential confounders. Our study, for the first time, provided intriguing evidence showing that non-obese NAFLD patients were prone to develop T2D in Chinese.

NAFLD is not rare in non-obese subjects. In the current study, the prevalence of NAFLD in a cohort of non-obese Chinese was 10.7%. The mechanisms of initiating and promoting NAFLD development in the absence of obesity are undetermined. Intra-familial aggregation (28) and inter-ethnic variations (29) in susceptibility suggest that genetic factors could be important in non-obese NAFLD. An analysis from the Dallas Heart Study found that a variant allele (rs738409) of *PNPLA3* was strongly associated with hepatic triglyceride content (30). Moreover, epidemiologic data suggest that the association was not mediated through obesity and insulin resistance (31–33). Interestingly, the at risk *PNPLA3* rs738409 GG genotype was found in 13–19% of the general population in Asian studies, compared with 4% in Caucasians, 2% in African Americans and 25% in Hispanics (30), displaying an ethnicity-related distribution pattern. A population-based study from Hong Kong showed that the *PNPLA3* polymorphism remained an independent risk factor for non-obese NAFLD after adjusting for other metabolic covariates (34). These data suggest that the *PNPLA3* variant could play an essential role in the pathogenesis of non-obese NAFLD. Emerging evidence also showed some other gene variants, such as *CETP* (35), *SREBF-2* (36), and *HLA* (37), associated with the development of non-obese NALFD. Genetic and mechanistic studies are warranted to dissect the contribution of those genetic variants to non-obese NAFLD.

Interestingly, studies showed a significant difference in the metabolic adaptation between patients with obese and non-obese NAFLD. Youngae et al. found that non-obese NAFLD and obese NAFLD subjects exhibited unique circulating lipidomic signatures, including diacylglycerol, triacylglycerol and sphingomyelin (38). Randy Levinson et al. showed a significant difference in blood bile acid, fibroblast growth factor 19 levels and gut microbiome between lean and non-lean NAFLD subjects (39). However, the causal association between these metabolic alterations and obese/non-obese NAFLD development is to be determined.

We found NAFLD was an independent risk factor for incident T2D in the non-obese Chinese population, suggesting a direct role of isolated NAFLD in T2D development in the absence of obesity. NAFLD is characterized by over-accumulation of lipids in hepatocytes. Excessive accumulation of lipid intermediates in the liver results in hepatic insulin resistance (40, 41), which might be a contributor to the increased T2D in non-obese NAFLD. Whether non-obese NAFLD has a similar risk of developing T2D relative to obese NAFLD is a matter of debate. A prospective cohort study from Sri Lanka showed no significant differences in the occurrence of new-onset metabolic comorbidities, including diabetes, between lean and non-lean NAFLD (42). However, another retrospective study from Italy reported that patients with lean NAFLD had a significantly higher risk of diabetes than non-lean NAFLD patients (43). In a large prospective cohort study from South Korea, Dong et al. found that the risk of incident diabetes is lower in lean participants with NAFLD than obese participants with NAFLD (44). More evidence is still needed to clarify the difference in diabetes risk between non-obese and obese NAFLD.

In addition to T2D, we found a higher prevalence of other established CVD risk factors, including dyslipidemia, hypertension, and smoking, in the NAFLD compared to the non-NAFLD group. Consistent with previous findings (45–47), dyslipidemia in non-obese NAFLD was mainly characterized by elevated TG and non-HDL-C levels and decreased HDL-C concentrations in this study. The pathogenesis processes of dyslipidemia in the setting of NAFLD are not fully understood. Although dyslipidemia in NAFLD could be attributable to coexisting obesity, an independent association between NAFLD and dyslipidemia was observed in non-obese subjects (47). Moreover, the association was stronger in non-obese than in obese individuals (47). These indicated the relationship between NAFLD and dyslipidemia independent of obesity.

Since tight associations between non-NAFLD and major CVD risk factors as mentioned above, it is unsurprising to find patients with NAFLD had a higher prevalence and incidence of CAD than those without NAFLD in the current study. However, after adjusting for other traditional CVD risk factors, the associations between non-obese NAFLD and CAD were lost both at baseline and during the 5-year follow-up period, even though a trend, not significant, of the association between non-obese NAFLD and incident CAD risk during follow-up, was observed (*p* = 0.059). Recent studies indicated dyslipidemia is the major contributing factor to the increased CVD risk in NAFLD patients (48, 49). These might explain the disappearance of the association between NAFLD and incident CAD risk in our study when adjusting factors including dyslipidemia. Moreover, prospective studies with longer follow-up duration are needed to further elucidate the long-term risk of CAD in non-obese NAFLD patients.

Our study has the strengths of large sample size with a relatively long follow-up observation. However, it also has several limitations. Initially, limited by examination methods, we did not assess liver tissue severity, including inflammation and fibrosis degrees, in patients with NAFLD, so that we could not determine the association of different forms of NAFLD with risk of T2D and CAD. Additionally, we did not measure blood insulin levels and evaluate the insulin resistance status of study participants. Lastly, this study is aimed to investigate whether the presence of NAFLD increases the risk of T2D and CAD in the absence of obesity. We did not include obese participants in this study. Therefore, we were not able to compare the risk of T2D and/or CAD in patients with non-obese NAFLD to those with obese NAFLD. Despite these limitations, our findings still provided important insights into the risk of T2D and CAD in non-obese NAFLD patients.

## Conclusions

NAFLD was an independent risk factor for T2D in non-obese subjects. However, no significant association was observed between non-obese NAFLD and incident CAD after adjusting other traditional cardiovascular risk factors, suggesting these factors might mediate the increased incidence of CAD in non-obese NAFLD patients.

## Data Availability Statement

The raw data supporting the conclusions of this article will be made available by the authors, without undue reservation.

## Ethics Statement

The studies involving human participants were reviewed and approved by the ethics committee institutional review board of The Second Xiangya Hospital of Central South University. The patients/participants provided their written informed consent to participate in this study.

## Author Contributions

SZ and WD: conceived and designed the study and wrote the paper. WD and ZZ: performed the study and analyzed the data. All authors read and approved the final manuscript.

## Conflict of Interest

The authors declare that the research was conducted in the absence of any commercial or financial relationships that could be construed as a potential conflict of interest.

## Publisher's Note

All claims expressed in this article are solely those of the authors and do not necessarily represent those of their affiliated organizations, or those of the publisher, the editors and the reviewers. Any product that may be evaluated in this article, or claim that may be made by its manufacturer, is not guaranteed or endorsed by the publisher.
