# Supplementary material for: The Risk of Type 2 Diabetes and Coronary Artery Disease in Non-obese Patients With Non-alcoholic Fatty Liver Disease: A Cohort Study
Source: Front Cardiovasc Med. 2021 Aug 20;8:680664. doi: 10.3389/fcvm.2021.680664 (PMC8417689; doi:10.3389/fcvm.2021.680664)
Supplement: Supplementary file 1 [file Data_Sheet_1.docx]

**Table S1. Incidence of T2D and CAD during follow-up.**

|  | Total | NAFLD | Non-NAFLD | p |
| --- | --- | --- | --- | --- |
| T2D, % (n) | 1.4 (177) | 4.8 (58) | 1.0 (119) | <0.001 |
| CAD, % (n) | 1.1 (134) | 2.3 (28) | 0.9 (106) | <0.001 |

Data were shown as percentage (number). P values from analyses of the chi-square test for comparisons between NAFLD and non-NAFLD group. Two-tailed p < 0.05 was considered statistically significant. NAFLD, Non-alcoholic fatty liver disease; T2D, type 2 diabetes; CAD, coronary artery disease.

**Table S2. Incremental values of NAFLD in predicting the risk of T2D.**

|  | C-statistic (95% CI) | ∆ C-statistic (95% CI) | p |
| --- | --- | --- | --- |
| Original model | 0.850 (0.830-0.871) | - | - |
| Model further added with the variable of NAFLD status | 0.860 (0.841-0.880) | 0.010 (0.002-0.020) | 0.015 |

Models were used to predict the risk of incident T2D. C-statistic and ∆C-statistic were used to evaluate the efficiency of models and the incremental predictive value of adding NAFLD status into original model. The Original model included variables of age, gender, body mass index and smoking. NAFLD, Non-alcoholic fatty liver disease; T2D, type 2 diabetes.

**Table S3. Cox proportional hazard regression analyses of the association of NAFLD severity with risks of T2D and CAD during follow-up.**

|  | Mild NAFLD  (n=1,064) | | | | Moderate to severe NAFLD  (n=152) | | | | |
| --- | --- | --- | --- | --- | --- | --- | --- | --- | --- |
|  | n* | HR | 95% CI | p | | n* | HR | 95% CI | P |
| Univariate analysis |  |  |  |  | |  |  |  |  |
| T2D | 50 | 4.6 | 3.3-6.4 | <0.001 | | 8 | 5.1 | 2.5-10.4 | <0.001 |
| CAD | 24 | 2.4 | 1.6-3.8 | <0.001 | | 4 | 2.9 | 1.1-7.8 | 0.039 |
| Multivariate analysis |  |  |  |  | |  |  |  |  |
| T2D^†^ | 50 | 2.4 | 1.7-3.3 | <0.001 | | 8 | 2.1 | 1.0-4.4 | 0.049 |
| CAD^‡^ | 24 | 1.5 | 1.0-2.5 | 0.071 | | 4 | 1.5 | 0.5-4.2 | 0.429 |

* Refers to the number of events of T2D and CAD. ^†^ Multivariate model adjusting for age, gender, body mass index and smoking for the HR of T2D; ^‡^ multivariate model adjusting for age, gender, body mass index, smoking, hypertension, low density lipoprotein cholesterol, high lipoprotein cholesterol, triglyceride and creatinine for the HR of CAD. The non-NAFLD group was set as the reference. P values from Cox proportional hazard regression analyses. Two-tailed p < 0.05 was considered statistically significant. NAFLD, Non-alcoholic fatty liver disease; T2D, type 2 diabetes; CAD, coronary artery disease; HR, hazard ratio; CI, confidence interval.
